# Frequency of Programmed Death Receptor Ligand 1 Expression and Clinicopathological Factors Associated With Metastatic Triple-Negative Breast Cancer at a Tertiary Cancer Care Centre in South India

**DOI:** 10.7759/cureus.55880

**Published:** 2024-03-10

**Authors:** Suresh Babu, Akansha Choudhary, Linu Jacob, Lokesh K.N., Rudresha A.H., Rajeev L.K., Smitha Saldanha, Usha Amirtham, Vijay C.R.

**Affiliations:** 1 Medical Oncology, Kidwai Memorial Institute of Oncology, Bangalore, IND; 2 Pathology, Kidwai Memorial Institute of Oncology, Bangalore, IND; 3 Epidemiology and Biostatistics, Kidwai Memorial Institute of Oncology, Bangalore, IND

**Keywords:** female cancer, aggressive cancer, south india, triple negative breast cancer, metastatic breast cancer, pdl1

## Abstract

Purpose

Triple-negative breast cancer (TNBC) has a poor outcome compared to other subtypes. Immune checkpoint inhibitors (ICIs) have changed the treatment paradigm in metastatic diseases as well as in neoadjuvant setting. The response to these agents is affected by programmed death ligand 1 (PDL1) receptor expression which are reported objectively as a score. PDL1 is a prognostic marker also. Here, we present clinicopathological characteristics of metastatic TNBCs, report the proportion of PDL1 expression and its association with clinicopathological factors as well as survival.

Methods

This is a prospective study carried out at a tertiary cancer care centre in South India. Case records of all breast cancer patients treated in two years between August 2021 and July 2023 were reviewed, patients with metastatic TNBC were selected. Patient’s characteristics, histological features, molecular profile, and treatment were analyzed. PDL1 testing was carried out on pretreatment tumor tissue sections with immunohistochemistry (IHC) (Dako 22C3). PDL1 staining was interpreted as negative or positive based on combined positive score (CPS), with an expression less than 10 considered negative.

Results

A total of 118 patients were analyzed. With a median age of 46 years (36-65 years), 52.5% (62/118) were premenopausal. Family history of Ca Breast was seen in 22% (26/118) patients. A majority of patients had left-sided tumor 55.9% (66/118). Visceral metastasis was more common 96.6% (82/118) than skeletal. Radical intent of treatment was adopted in 10% as patients had oligometastatic disease at presentation. As front-line treatment, anthracycline-based chemotherapy was administered to the majority 54.2% (64/118). The PDL1 expression with CPS more or equal to 10 was seen in 32.2% (38/118) patients. Survival was associated with menopausal status (p value=0.000) and family history (p value=0.028) but not with PDL1 nor sidedness in our study. Estimated survival at 12 months in PDL1 negative case is 10 ± 0.29 months, while in PDL1 positive case it is slightly more at 10 ± 0.75 months, but difference was not found to be statistically significant (p value=0.15).

Conclusion

TNBCs are highly aggressive subtype with limited treatment options and poorer outcomes. Our study shows PDL1 expression in 31.66% of the cases similar to other literature from India. Survival is associated with menopausal status and family history. No association was found between survival and PDL1 as well sidedness in our study.

## Introduction

Globally, breast cancer is the most common cancer among females in terms of incidence as well as mortality. In India, it is the most common cancer, accounting for 25% of all cancers [1,2]. Triple-negative breast cancers (TNBC) accounts for nearly 15% of total breast cancer cases worldwide, however, in low- and middle-income countries including India (LMIC) it is almost three in every 10 cases [3-5]. TNBC is more common among young females. Due to their aggressive biology and the early visceral metastases that are a part of TNBC's natural history, these women have low survival rates [6,7]. Chemotherapy has long been the only available treatment for this subgroup. Patients with programmed cell death ligand 1 (PDL1)-expressing tumors having advanced TNBC can now get immunotherapy in addition to chemotherapy. Relapse is frequent and usually happens early, despite the high response rate of TNBC to chemotherapy [4]. TNBC is incurable once metastasis develops, with a median overall survival (OS) of around a year [8].

At the molecular level, TNBC is a group of breast tumors with diverse pathology. Transcriptome analyses have reclassified breast tumors into intrinsic subtypes, such as normal breast-like, luminal A and luminal B (Estrogen Receptor (ER) + and/or Progesterone Receptor (PR) +), Human Epidermal Growth factor receptor 2 (HER-2) enriched, claudin-low, and basal-like, following the American Society of Clinical Oncology/College of American Pathologists (ASCO/CAP) classification [8,9]. While breast tumors with low levels of ER and PR expression (1-10%) are more likely to be luminal (46%) or HER-2-enriched (29%) by gene expression, the vast majority of ASCO/CAP-defined TNBCs (50-75%) are basal-like [10]. The majority of breast cancers with minimal claudin expression lack immunohistochemistry (IHC) expression of ER, PR, and HER-2 and exhibit metaplastic/medullary differentiation on histology. They are also linked to active transforming growth factor-beta signalling, stem cell and mesenchymal characteristics, and elevated expression of genes relevant to the immune system. As a result, the basal-like and claudin-low breast cancer subtypes, identified by gene expression criteria, and TNBC, as determined by IHC criteria, are not interchangeable. Compared to other types, TNBC exhibits a higher frequency of intra-tumor lymphocytes and the expression of PDL1. Transmembrane protein PDL1 is present in many different types of cells, including neoplastic cells [11,12]. Once active, the PD-1/PDL1 signaling pathway allows tumor cells to evade the immune system [11,13]. The quantification of PDL1 is done as a biomarker to forecast the effect of immuno-oncology drugs.

The results reported by the sparse available literature regarding the PDL1 expression in breast cancer are varied due to the use of different antibody clones, various scoring system, evaluation in lymphocytes and neoplastic cells [13-15]. There are only a few studies on PDL1 expression in breast cancer. However, patients with metastatic TNBC have been missing or not appropriately represented in these studies. Therefore, the present study was carried out with an aim to measure PDL1 expression in metastatic TNBC and study clinical, pathological and survival characteristics in the same population. A study focussing on PDL1 in metastatic TNBC has never been carried out before. The study was carried out and survival was measured without the use of immune checkpoint inhibitors (ICIs), due to resource constraint settings. It is a rapidly evolving area wherein ICI therapy with atezolizumab was first approved and then withdrawn. Later pembrolizumab has been used in metastatic and subsequently in neoadjuvant setting as well [16-18].

## Materials and methods

This is a prospective study carried out in the Department of Medical Oncology at a tertiary cancer care centre in South India after obtaining ethical approval from the Medical Ethics Committee (Institutional Review Board) affiliated to Kidwai Memorial Institute of Oncology. Informed consent of the patients was taken. Case records of all breast cancer patients treated with chemotherapy in two years between August 2021 and July 2023 were reviewed patients with metastatic TNBC were selected. The study included adult female breast cancer patients with histologically confirmed TNBC and metastatic disease. Patients with metastatic disease who were previously treated for limited stage as well as newly diagnosed metastatic TNBC were prospectively followed. Male breast cancer, those aged <18 years and those not willing to give informed consent were excluded. Patient’s demographic details, tumor characteristics, morphological features, details of IHC like ER, PR, HER-2 etc. and treatment given were studied. PDL1 testing was carried out after the patient developed metastasis. For PDL1 testing, IHC using 22C3 clone from manufacturing Company Dako Agilent was used.

Staging was done with either bone scan and contrast-enhanced CT (CECT) or positron emission tomography-CT (PETCT). Patients were staged according to American Joint Committee on Cancer (AJCC)-7 tumor node metastasis (TNM) staging system. TNBC was defined as ER negative, PR negative, and HER-2 neu negative cancers. IHC tests were carried out with standard Food and Drug Administration approved kits. For each patient, antibody staining of a set of paraffin embedded slides for ER, PR and HER-2 was carried out.

A HER-2 score of 3 or higher on an IHC tumor was regarded as positive. A fluorescence in situ hybridization test was performed on those patients who had an IHC score of HER-2 neu 2+. ASCO/CAP standards deemed a HER-2 score of 0 or 1 on IHC to be negative. Any breast cancer that showed signs of distant metastasis was classified as metastatic breast cancer (MBC). Using the Dako 22C3 IHC test, the expression of PDL1 was investigated in tumor cells and immune cells. The combined positive score (CPS) is calculated by dividing the number of PDL1 positive cells, including tumor cells, lymphocytes, and macrophages by the total number of viable tumor cells which is further multiplied by 100.

Descriptive analysis was done for the baseline characteristics. T test (continuous variables) and a chisquare test (categorical variables) were used to compare patient characteristics. Survival was compared using Kaplan-Meier curve and statistical significance was analyzed using log-rank test. SPSS version 29 (SPSS Inc, IBM, United States) was used for statistical analysis. The institutional ethics committee gave its approval to the project. Case records were examined, informed consent was taken for conducting the study as well as publication. It was a prospective study. Since none of the patient identities were disclosed in the data, confidentiality was preserved. The protocols adhered to the 2013 revision of the Helsinki Declaration of 1964 and the ethical guidelines set forth by the competent committee on human testing.

## Results

During the study period of two years, a total of 968 breast cancer patients were registered at our institute and out of these 208 (21.48%) were TNBC. Further out of 208 TNBC breast cancer patients 57.69% (120/208) patients were metastatic. Case records were not available for two patients. The data of 118 patients was analyzed for the study. Median age of presentation was 46 years with range of 36-65 years. while the majority were premenopausal 52.5% (Table 1). More women (76.2%; 92/118) hailed from rural background than urban. The median age of attaining menarche was 14 years. The median age at first pregnancy was 26 years with minimum age being 16 years and the maximum 35 years, except four women who were nulliparous.

**Table 1:** Baseline characteristics IDC: Invasive ductal carcinoma

Variables	Frequency
Median age	46 years (36-65 years)
Menopausal status	
Premenpausal	62 (52.5%)
Postmenopausal	56 (47.5)
Family history of cancer	26(22%)
Sidedness	
Left-sided tumor	66 (55.9%)
Right-sided tumor	52 (44.1 %)
Sites of metastasis	
Visceral	82 (96.6%)
Bone	48 (56.6%)
Histology	Frequency
IDC	110 (93.2%)
Metaplastic	2(1.7%)
Medullary	3 (2.5%)
Grade I	0 (0%)
Grade II	5 (2.5%)
Grade III	115 (97.5%)

**Figure 1:**
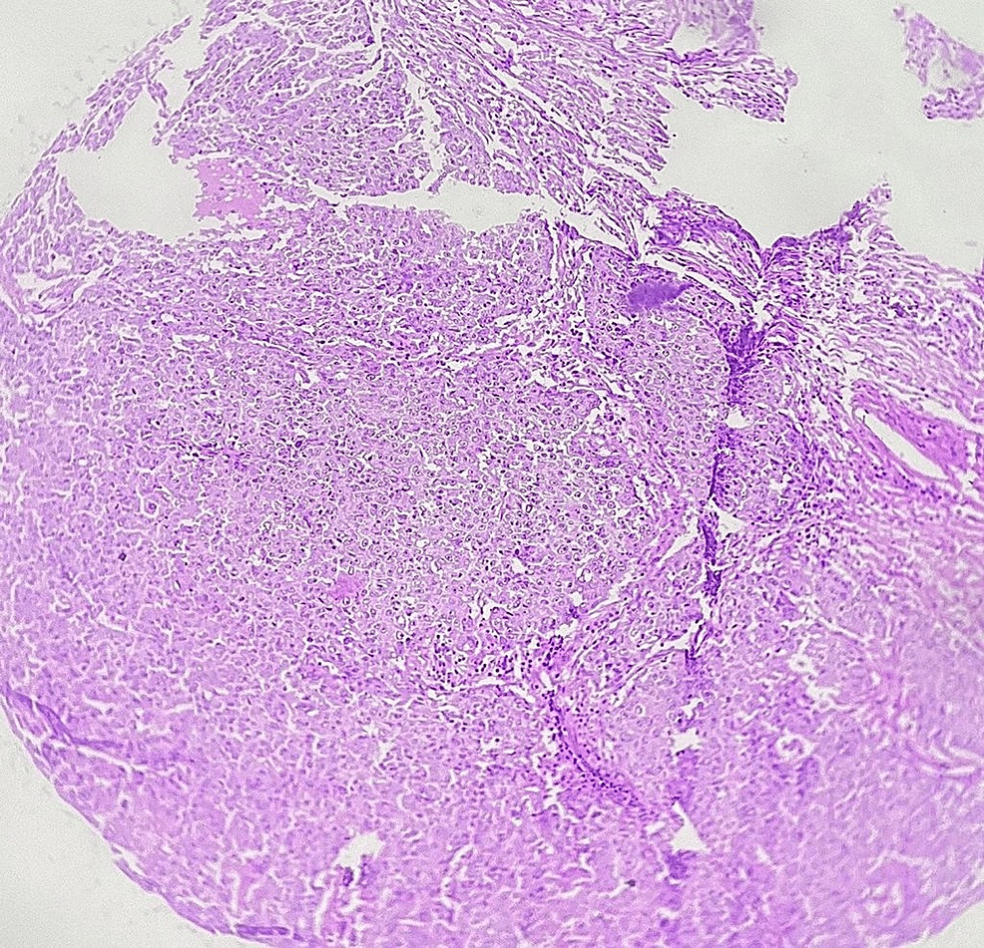
Histopathologic examination depicting neoplastic cells in low-power view under the microscope

The most common presenting complaint was lump in breast. Left side (55.9%; 66/118) was more common than the right side. The average number of children was 2.1.Family history of breast cancer was seen in 22% (26/118) patients. The most common histological subtype in our study was that of infiltrating ductal carcinoma, not otherwise specified (NOS), similar to other studies as seen in Figure 1 [14,15]. Other subtypes found included medullary in three patients, and metaplastic differentiation was seen in another two.

Visceral metastasis was most common (96.6%; 82/118), moreso than skeletal (56.6%; 48/118). Radical intent of treatment was adopted in 10% as these patients had oligometastatic disease at presentation. As first line of chemotherapy, anthracycline-based chemotherapy was administered to the majority (54.2%; 64/118). Taxane-based chemotherapy was administered to 37.2% (44/118). Anthracycline- and taxane-based chemotherapy was administered to 10.1% (12/118) who were planned for radical intent. The PDL1 expression with IHC using a CPS more than or equal to 10 was seen in 32.2% (38/118) patients. The difference between PDL1 positive and negative tumors is depicted in Table 2.

**Table 2:** Showing differences between PDL1 expression positive and negative metastatic TNBCs PDL1: Programmed death receptor ligand 1; TNBC: Triple-negative breast cancer

	PDL1 negative	PDL1 positive	P-Value
No. of patients	80	38	
Mean age (years)	48.4 ± 11	49.3 ± 17.4	0.655
Age group (years)			
<40	22 (27.5%)	4 (10.5%)	1
40-50	31 (38.8%)	21 (55.3%)	
50-60	13 (16.3%)	7 (18.4%)	
>60	14 (17.5%)	6 (15.8%)	
Ki67 index	50.3 ± 21.2	59.2 ± 17.4	0.027
				Menopausal status			
Premenopausal	45 (36.3%)	17 (44.7%)	0.3
Postmenopausal	35 (43.87%)	21 (55.3%)	
Ki67 index			
<20	17 (21.3%)	3 (7.9%)	0.016
20-40	46 (57.5%)	18 (47.4%)	
40-60	5 (6.3%)	9 (23.7%)	
>60	12 (15.0%)	8 (21.1%)	
Sidedness			
Left sided	45 (56%)	21 (55%)	1
Right sided	35 (43.8%)	17 (43.7%)	
Family history			
Absent	62 (77.5%)	30 (78.9%)	1
Present	18 (22.5%)	5 (21.1%)	

**Figure 2:**
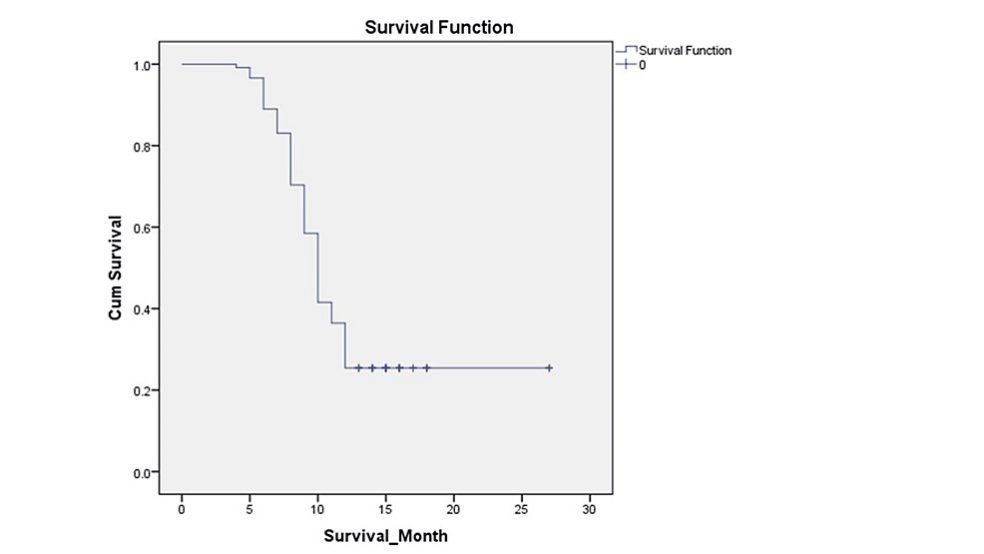
Kaplan-Meier curve of all metastatic TNBC patients at one year TNBC: Triple-negative breast cancer

At 12 months, 88 events occurred, and 30 were censored. The Kaplan-Meier curve depicts survival at 12 months in Figure 2. Median survival was 10 months. Survival was associated with menopausal status (p value=0.000) and family history (p value=0.028). No association could be demonstrated between survival and PDL1 nor between survival and sidedness in our study. Estimated survival at 12 months in PDL1 negative case is 10 ± 0.29 months, while in PDL1 positive case it is slightly more at 10 ± 0.75 months, but not statistically significant (p value=0.15).

## Discussion

Despite its little percentage, TNBC makes a significant contribution to breast cancer morality and is a topic of current research worldwide. We looked on the prevalence of PDL1 in metastatic TNBC along with patients’ demographic details, tumor characteristics, morphological features, IHC, and treatment given. The incidence of TNBC in the current study was 32.2% (38/118) patients. As with previous research, infiltrating ductal carcinoma (NOS) was the most prevalent histological subtype in our investigation [19-22]. In our study, 31.66% (38/120) of the patients had tumor PDL1 expression. The percentage ranges from 30% to 60% in the scant literature, that is currently accessible [19,20]. Various Indian studies show the PDL1 proportion ranging from 13% to 59% [23-26]. A study by Mehan et al. from North India showed the proportion of PDL1 positivity as 59.2% in immune cells while 37.9% in tumor cells [21]. Sharma et al., also from the northern part of India, reported PDL1 to be positive in 32.5% of the cases in tumor cells and found no correlation between PDL1 positivity in tumor cells and tumor infiltrating lymphocytes positivity [22]. Another study from the west of India reported PDL1 to be 31% [23]. Bhardwaj et al. reported PDL1 to be positive in 52% of immune cells and 13% of tumor cells among patients treated in South India [24]. Darga et al. determined the positivity of PDL1 in circulating tumor cells and platelets in patients with MBC in United States as 40% and 28% respectively [25]. In a recent study reported from North India by Punahani et al., PDL1 was positive in 14.67% of TNBCs [26]. Table 3 shows a comparison of various studies on frequency of PDL1 in breast cancers from different parts of India.

**Table 3:** A comparison of various studies on frequency of PDL1 in breast cancers from different parts of India PDL1: Programmed death receptor ligand 1; TNBC: Triple-negative breast cancer

Study	Present Study	Bhardwaj et al. [24]	Ghosh et al. [23]	Sharma et al. [22]	Mehan et al. [21]	Punhani et al [26].
1. Region of India	South India	South India	East India	North India	North India	North India
2. Year of publication	-	2019	2021	2021	2022	2023
3. Number of patients	120	132	107	40	103	150
a. Metastatic disease	120	NA	Nil	20	14	NA
b. Non-metastatic disease	-	NA	107	-	89	NA
4. Molecular classification	TNBC	All including TNBC	TNBC	TNBC	All subtypes	All subtypes
5. Median age	46	NA	Ventana SP 142	40 to 60	51	NA
6. Platform used	22C3	RTqPCR	-	-	SP 263	NA
7. PDL1 Positivity	31.66% in tumor and immune cells	52% in immune cells 13% in tumor cells amongst TNBC	NA	32.5% in tumor cells	59.2% in Immune cells 37.9% in tumor cells	14.67

Median survival was 10 months in our study. Survival was associated with menopausal status (p value=0.000) and family history (p value=0.028). No association was found between survival and PDL1 as well as sidedness. Estimated survival at 12 months in PDL1 negative case is 10 ± 0.29 months, while in PDL1 positive case it is slightly more at 10 ± 0.75 months, but not statistically significant. (p value= 0.15). It is not very clear if PDL1 positive status is associated with poor or improved survival as various studies have yielded different results. According to prior research on breast cancer by Botti et al. and Schalper et al., PDL1 expression was associated with improved disease-free survival (DFS) but had no effect on OS [11,27]. However, a few metanalyses suggest worse OS and no association with DFS [28-30].

A major limitation of the previous studies from India is that they have been carried out in the department of pathology, so the patient characteristics were not analyzed in detail and with respect to clinical characteristics. As per our knowledge, PDL1 status was not analyzed with respect to survival in India by any previous studies. The results amongst metastatic TNBC were not so accurate as hormone receptor positive cases and HER-2 positive cases were also included. Apart from this, about extent of disease, majority of patients had locoregional disease rather than being metastatic. Also, the platform used in most of the studies was SP 142 essay, which has limitations in the form of percentage positivity. Atezolizumab, for which SP 142 was approved as the companion diagnostics, has been withdrawn, while pembrolizumab with Dako 22C3 as companion diagnostics is approved in metastatic setting and neo adjuvant setting as well. Our study has limitations being a single-centre study and small sample size. No ICI or any other treatment based on PDL1 status was given in our study due to resource constraint settings.

## Conclusions

Among the patients with metastatic TNBC, PDL1 expression in immune cells was seen in 32.2 % at our centre. Median survival was 10 months in our study. Survival is associated with menopausal status and family history. Premenopausal women and those with positive family history have poor survival. Association between survival and PDL1 or sidedness was not demonstrated in our study, in the absence of use of ICIs. For pembrolizumab therapy in patients with metastatic TNBC, PDL1 testing is needed with Dako 22C3 companion diagnostics, whereas atezolizumab, which required PDL1 testing with Ventana SP 142, has been withdrawn. PDL1 testing is not required for localized breast cancer in neo adjuvant setting. As the field of ICI therapies and the role of PDL1 assays/scores is dynamic, there should be more studies in this area. On IHC, the assessment of PDL1 status should be reported and therapy should be administered accordingly as it leads to improved survival. As ICI usage is going up, due to increased availability, it is important to have baseline data on PDL1 in TNBC. There may be differences in percentage of PDL1 positivity in Western and Indian population, hence more studies are needed in this field.
